# SARS-CoV-2 RNA Persistence in Naso-Pharyngeal Swabs

**DOI:** 10.3390/microorganisms8081124

**Published:** 2020-07-26

**Authors:** Maria Luisa Danzetta, Laura Amato, Francesca Cito, Alessandra Di Giuseppe, Daniela Morelli, Giovanni Savini, Maria Teresa Mercante, Alessio Lorusso, Ottavio Portanti, Ilaria Puglia, Federica Monaco, Claudia Casaccia, Annapia Di Gennaro, Lilia Testa, Giacomo Migliorati, Nicola D’Alterio, Paolo Calistri

**Affiliations:** Istituto Zooprofilattico Sperimentale dell’Abruzzo e del Molise G. Caporale, 64100 Teramo, Italy; l.amato@izs.it (L.A.); f.cito@izs.it (F.C.); a.digiuseppe@izs.it (A.D.G.); d.morelli@izs.it (D.M.); g.savini@izs.it (G.S.); t.mercante@izs.it (M.T.M.); a.lorusso@izs.it (A.L.); o.portanti@izs.it (O.P.); i.puglia@izs.it (I.P.); f.monaco@izs.it (F.M.); c.casaccia@izs.it (C.C.); a.digennaro@izs.it (A.D.G.); l.testa@izs.it (L.T.); g.migliorati@izs.it (G.M.); n.dalterio@izs.it (N.D.); p.calistri@izs.it (P.C.)

**Keywords:** SARS-CoV-2, coronavirus, qPCR, Abruzzo, COVID-19, naso-pharyngeal swab, viral shedding

## Abstract

Since February 2020, Italy has been seriously affected by the SARS-CoV-2 pandemic. To support the National Health Care system, naso-pharyngeal/oropharyngeal swabs collected from suspected cases of Teramo province, Abruzzo region, are tested at Istituto Zooprofilattico Sperimentale dell’Abruzzo e del Molise G. Caporale, for the presence of SARS-CoV-2 RNA. Out of 12,446 tested individuals, 605 returned positive results at least once, with prevalence significantly higher in men. A reduction of the level of viral RNA in the first swab per each positive patient collected over time was also observed. Moreover, 81 patients had at least one positive sample and two final negative tests: positivity in swabs lasted from 14 to 63 days, with a median value of 30 days. This shows the potential for the virus to coexist with patients for a long time, although we highlighted intermittent positivity in several cases. The evolution of the SARS-CoV-2 epidemiological situation and knowledge on viral shedding should be closely monitored, to interpret the findings correctly and adjust accordingly the surveillance activities.

## 1. Introduction

Eighteen years after the emergence of severe acute respiratory syndrome (SARS) in China, and eight years after the emergence of Middle East respiratory syndrome (MERS) in Saudi Arabia, a novel coronavirus (CoV) epidemic of a probable animal origin, acknowledged as a pandemic on March 11 2020 by the WHO [1], is threatening the human population worldwide [2].

The disease, referred to as coronavirus disease 2019 (COVID-19) [3], is caused by a novel human CoV named SARS coronavirus 2 (SARS-CoV-2) [4,5]. This virus belongs to the species *Severe acute respiratory syndrome-related coronavirus* (*SARS-rCoV*), sub genus *Sarbecovirus*, genus *Betacoronavirus*, sub family *Orthocoronavirinae*, family *Coronaviridae*, with other known viruses mainly identified in humans, wild carnivores, and bats [5,6].

The virus was first identified in Wuhan, Hubei province of China, where pneumonia cases of unknown origin were observed since mid-December 2019 [2]. Despite China and many other countries promptly placing various control measures [7], sustained human-to-human transmission of SARS-CoV-2 occurred rapidly outside Wuhan [8] and by the end of January 2020, about 20 other countries reported COVID-19 cases internationally [9]. Since the emergence of SARS-CoV-2, more than 6.2 million COVID-19 cases were reported globally [10] (up to the 3rd of June). Italy is one of the most heavily hit countries in Europe: as of the 3rd of June, more than 233,500 cases overall were confirmed, with 33,530 reported fatalities [11]. Italy was one of the first Western countries to report an alarming rise of COVID-19 cases, and on the 23rd of February the Italian Government imposed a lockdown in the two hotspot areas, in northern regions of Lombardy and Veneto [12]. On March 9th, in response to the growing number of cases reported, emergency measures based on the restriction of human mobility, social distancing, and closure of all non-essential services were extended to the whole country [13].

In Abruzzo, a region located in the central part of Italy, the first case of COVID-19 was recorded in a male patient on 27 February [14]. The patient originated from Lombardy region, and arrived in Abruzzo for tourism some days before the movement restrictions were implemented all over the Italian territory [14]. Up to the 3rd of June 2020, 3252 COVID-19 cases were confirmed in Abruzzo [15].

In order to support the National Health Care system, the Ministry of Health appointed the Istituti Zooprofilattici Sperimentali (IZSs), which are public veterinary institutes, to test the naso-pharyngeal swabs collected from suspected human cases for SARS-CoV-2 [14]. The swabs were collected based on clinical symptoms or reported contact with confirmed COVID-19 cases. Therefore, understanding the nature of the test used, and interpreting the results and how these may vary over time in samples collected from the same patient, is of paramount importance [16]. In general, a “positive” molecular result may reflect only the detection of viral RNA and does not necessarily the presence of viable virus [16,17,18]. Nevertheless, a patient with a respiratory tract specimen positive to SARS-CoV-2 RNA may represent an infectious source of COVID-19 [19] and, for the principle of precaution, proper preventive measures must be applied.

In addition to a wide overview of the results originating from SARS-CoV-2 diagnostic activities performed at IZSAM in the last 3 months, this paper mainly focuses upon SARS-CoV-2 RNA persistence in naso-pharyngeal/oropharyngeal swabs.

## 2. Materials and Methods

### Ethical Approval Statement

The testing of suspected COVID-19 cases and contacts in Teramo province was conducted within the official surveillance programme established by the Italian Health Authorities and did not require ethical approval.

Starting from February 29th, naso-pharyngeal/oropharyngeal swabs collected from suspected cases of Teramo province, Abruzzo region, are tested at IZSAM for the presence of SARS-CoV-2 RNA. Samples were collected from the respiratory tract of individuals either hospitalized, or screened as having a contact history with infected individuals, or in the framework of screening programs for health workers belonging to the National Health Care System. 

Two steps compose the workflow for SARS-CoV-2 RNA detection. The first includes virus inactivation (PrimeStore^®^ MTM, Bethesda, MD, USA) in BSL3 bio containment laboratory, starting from a total volume of 200 μL oropharyngeal swab transport medium (physiological solution) or bronchoalveolar lavage (BAL) and nucleic acid purification (MagMaxTM CORE, Thermo Fisher Scientific, Waltham, MA, USA), according to the manufacturer’s instructions. The second consisted of RNA detection by the TaqMan^TM^ 2019-nCoV Assay Kit v1/v2 (Thermo Fisher, qPCR), whose results are interpreted following the manufacturer’s instructions. Specifically, this test targets three different portions of SARS-CoV-2 genome located in the replicase, the S and the N protein encoding genes, respectively [14]. For practical reasons in the downstream analysis, out of the three C_T_ values produced for each diagnostic target, we selected the C_T_ value associated with detection of the N protein encoding gene, since it is translated by the most abundant viral subgenomic RNA [20].

In the present study, we took into consideration samples from Teramo province collected and tested at IZSAM facilities in the interval between February 29th and May 21th for descriptive statistical analysis. A chi-square test was used to assess statistical significance between categorical variables (positive/negative patients) of the two groups (males and females). A two-tailed Mann–Whitney test was applied to compare the two groups: positive/negative and age of each patient. In addition, a Mann–Whitney test was used to assess statistical significance among the C_T_ N gene median values among the months from March to May. To this end, for each patient testing positive, the C_T_ N gene value at the first positive swab was considered.

Amongst the positive patients, we selected those who returned positive results at least twice, as a cohort, to study the duration of RNA positivity in swabs. A pairwise correlation test was performed to evaluate the correlation between the age of patients, the duration of RNA positivity in swabs, and C_T_ values for N protein encoding gene. In addition, a two-tailed Mann–Whitney test was applied to compare the median C_T_ values at the first sample and gender. Finally, a Wilcoxon signed rank test was used to assess significance in the trend of C_T_ gene values over time until the first negative swab.

The statistical analysis was performed using StatTools© (version 7.5.2 Palisade Corporation, Ithaca, NY, USA). The level of significance was set at a *p*-value of 0.05.

## 3. Results

### 3.1. qPCR Results and Data Analysis

**Figure 1:**
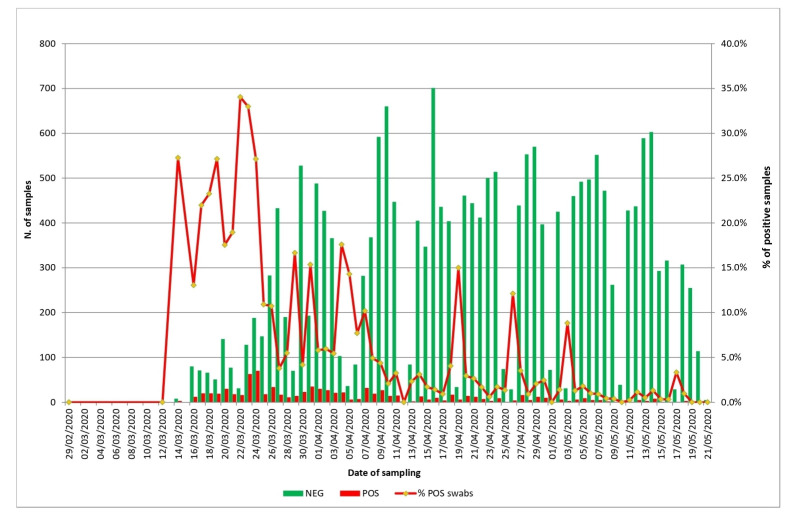
Temporal distribution of samples tested by results, and percentage of positive samples.

From February 29th to May 21th of 2020, IZSAM analyzed a total number of 20,878 naso-pharyngeal swabs collected from 14,200 individuals in Teramo province: 854 swabs tested positive by qPCR (Figure 1) naso-pharyngeal.

Information about gender was available for 12,446 out of 14,200 patients. Taking into consideration these 12,446 patients, 43.7% were males (*n* = 5,440) and 56.3% females (*n* = 7,006). Moreover, 605 tested positive at least once, and 11,841 were negative. Within the group of positives, 52.2% (*n* = 316) were male and 47.8% (*n* = 289/605) were females. The difference observed between genders’ prevalence of infection was statistically significant (X^2^ = 18.772 *p*-value < 0.0001). Positive patients represented 5.8% of the male group (95% IC 4.9–6.5%) and 4.1% of the female patients (95% IC 3.7–4.6%).

Information about age was available for 14,016 patients, 626 of which tested positive. The mean age of positive and negative individuals was compared: mean age of negatives was 53.4 years (±20.3 standard deviation), whereas positive individuals were 55.0 years on average (± 20.3 standard deviation). The difference between the mean age of positives and negatives was statistically significant (two-tailed Mann–Whitney statistical test: *p*-value < 0.0001).

### 3.2. RNA Persistence in Swabs

**Figure 2:**
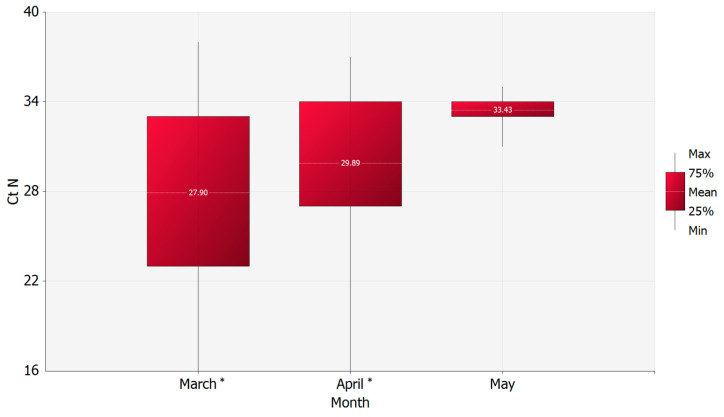
Mean values of CT N gene of the first positive swab per patient by month. * Asterisks are denoting the significant mean value of CT N gene (*p*-value = 0.0002).

**Figure 3:**
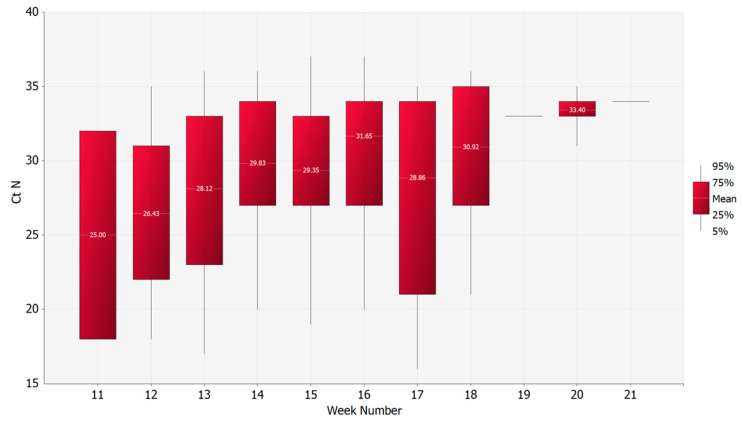
Trend of C_T_ N gene mean values of the first positive swab per patient by week of sampling.

The C_T_ N gene values of the first swab from each of the 605 positive patients where gender was available were compared over time. The mean C_T_ N gene values of those collected in March were statistically significantly lower than those collected in April. This suggests higher mean viral loads in March compared to April, as the C_T_ N gene values are inversely proportional to viral loads (two-tailed Mann–Whitney statistical test: *p*-value 0.0002). However, no statistically significant difference was found between April and May (two-tailed Mann–Whitney statistical test: *p*-value 0.0732) (Figure 2). Moreover, a correlation between the C_T_ N gene values and the week of sampling was found (*p*-value = 0.0001, Pearson’s correlation coefficient = 0.218) (Figure 3).

**Figure 4:**
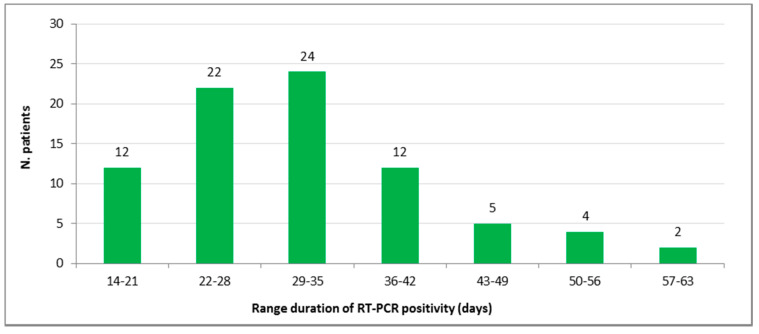
Number of patients distributed according to the range duration of qPCR positivity (Min = 14; Max = 63).

Between March 14th and May 6th, 108 patients tested positive by qPCR more than once. Therefore, they were followed up on until June 3rd for calculation of SARS-CoV-2 RNA persistence in swabs. Up to June 3rd, 81 of the initial 108 patients had a double negative final naso-pharyngeal/oropharyngeal swab, thus indicating a possible recovery from the infection. The difference in days between the date of the first qPCR positivity and the first negative swab in individual patients varied from 14 to 63 days, with a median value of 30 days (± 10.68 days of standard deviation) (Figure 4).

**Figure 5:**
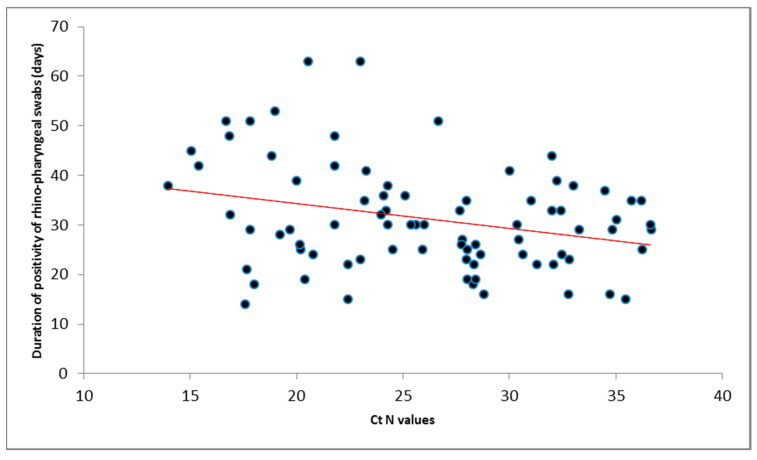
Scatterplot of C_T_ N gene values at the first positive swab and the SARS-CoV-2 RNA persistence in naso-pharyngeal/oropharyngeal swabs per patient.

There was a negative correlation between the C_T_ N gene values at the first positive swab and duration of RNA persistence, suggesting higher viral loads were correlated with longer duration of viral shedding (*p*-value = 0.009 Pearson’s correlation coefficient = −0.289) (Table 1, Figure 5). No correlation was observed between either the age of the patients and C_T_ N gene values or the duration of RNA persistence in swabs.

**Figure 6:**
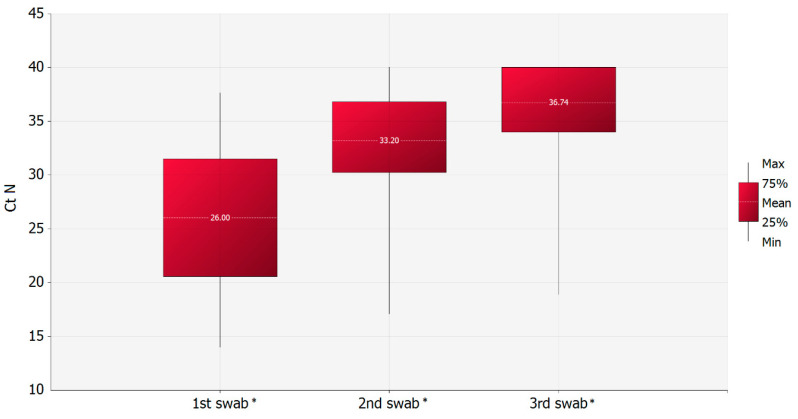
Time variation of the CT N gene mean values of patients with final double negative swab. * Asterisks are denoting the significant mean values of CT N gene (*p*-value = 0.0001).

Of the 81 patients with a double negative final swab, the mean value of the C_T_ N gene in subsequent swabs of the same patient, was significantly higher than the previous one (Wilcoxon Signed Rank test for paired samples: *p*-value = 0.0001, Z = 6.3570 between 2nd and 1st swab, Z = 5.4522 between 3rd and 2nd swabs) (Figure 6).Of the same group of patients, 38 were females and 43 males. For females, the range duration of qPCR positivity was between 14 and 51 with a median value of 29.5 (± 8.6 days of standard deviation), while for men the range varied between 15 and 63 days with a median value of 30 (± 12 days of standard deviation). The difference between males and females of the mean number of days during which the positivity of swabs persisted was found not statistically significant (two-tailed Mann–Whitney statistical test: *p*-value 0.2290). Additionally, the difference between genders and C_T_ N gene values at the first positive swab sample was not significant (two-tailed Mann–Whitney statistical test: *p*-value 0.3204). However, when the correlation test between the C_T_ N gene values at the first positive swab sample and the RNA persistence was performed separately for males and females, a significant correlation was observed only in males (*p*-value = 0.022, Pearson’s correlation coefficient = −0.349) (Table 1). 

## 4. Discussion

Bearing in mind that among the 605 positive patients for which the gender was known, 52.2% were male, we found that the SARS-CoV-2 prevalence in men was significantly higher compared to women. The role of gender in morbidity and mortality of SARS-CoV-2 infection was investigated by Jin et al. [21], who determined gender as a risk factor for severity and mortality in patients with COVID-19 in China [21]. In this regard, we were not able to analyze the mortality rate by gender owing to the lack of follow-up information on the ongoing health status of the patients considered in the study. In the analyzed samples, SARS-CoV-2 RNA was identified in patients belonging to all age groups. Overall, the highest percentage of SARS-CoV-2 RNA positive cases occurred in the age group of 50–59 years; this class was also the most represented in terms of number of people sampled, for both females and males. However, considering also the gender, most cases involving men were in the 60–69 years age class, while for positive females, the most represented age class was the 50–59 years group.

Depending on the collected specimen, the timeline of qPCR positivity may vary: qPCR positivity declines slower in sputum, and may still be positive after naso-pharyngeal swabs are negative [16,18]. Many coronaviruses can also be transmitted through the oral–fecal route, and patients may still be shedding virus through other routes, but return a negative oral/nasal swab [22]. In our study, only naso-pharyngeal/oropharyngeal specimens were collected and analyzed; therefore, it was not possible to compare the results of different specimens collected from the same patient.

Interestingly, when levels of C_T_ N gene values in positive naso-pharyngeal swabs were compared over time, a reduction of the level of viral RNA was observed in swabs collected in April when compared to those collected in March. In contrast, the absence of a clear statistical difference between May and April was seen (*p*-value 0.0732), mainly due to the low number of positive samples collected in May (*n* = 7). A reduction trend of viral RNA levels in naso-pharyngeal swabs was observed across time, which is difficult to explain in light of the lack of consistent viral mutations of potential clinical or pathological relevance [23] or, alternatively, of mutations affecting the efficiency of the molecular test. Moreover, it should be noted that, despite the lack of a standard curve for the absolute quantification of samples, the reduction trend was observed also for the other two genome targets of the selected molecular assay.

The information about the clinical status of all positive persons was not available. Consequently, we can only hypothesize that either the surveillance increased over time, including testing of persons at risk and in significant contact with confirmed cases, or the elimination of infection foci over time was able to reduce the general viral load in the exposure environments (for example households, hospitals, and other communities). Further investigations are needed to confirm and better explain our findings, including the collection of more data for each positive individual, relevant to the clinical status and the possible way of exposure.

Regarding the duration of viral shedding, a study previously performed in Wuhan reported a median duration of viral shedding in symptomatic patients of 20 days (range 8–37) [24]. In another study [25], a shorter median duration of viral shedding was described (12 days, range 4–34). Only single cases of longer duration have been identified so far: 49 days and 72 days, in two separate studies [26,27]. We observed a longer mean duration of qPCR positivity, ranging from 14 to 63 days (mean = 29; Std. Deviation = 9.89) in the 81 cases with final double negative naso-pharyngeal/oropharyngeal swabs. The difference between values of the duration of viral RNA positivity as well as the C_T_ N gene values at the first positive swab sample between males and females was not statistically significant, in contrast to another study in which the virus was identified for a significantly longer period in men than in women [28]. However, the observation of a stronger correlation in males between the duration of viral RNA persistence in naso-pharyngeal/oropharyngeal swabs and C_T_ N gene values at first sampling, seems to indicate a role of gender in the persistence of the infection. It appears that SARS-CoV-2 virus has the potential to coexist with patients for a long time, with serious implications for prolonged required quarantines and global efforts to control the infection [27]. However, it should be remembered that the detection of viral RNA does not indicate the presence of viable virus [18].

Two out of the 81 patients had two double negative naso-pharyngeal/oropharyngeal swabs before having one more positive sample, and then finally turning negative (double swabs) again. Respiratory shedding may be intermittent; therefore, a single negative swab could be misleading [29]. A few similar cases have already been reported, but uncertainty persists over what could have been a testing error, reinfection, or reactivation of the virus [16,27]. In our case, one of the two patients was hospitalized, while the other was assisted at home by the Health Care System. In the first case, dyspnea was reported, while the other experienced coughing.

Lastly, for 27 patients out of the 108 selected as a cohort, it was not possible to estimate SARS-CoV-2 RNA persistence in swabs because of the absence of the double negative swab in our records. A further limitation of this study was the lack of information on the clinical status for each qPCR positive case: we had partial or inconsistent information on the presence or absence of symptoms, therefore an important part of the picture is missing, especially in the interpretation of the cases with conflicting results. For 10 out of these 27 patients, a fluctuation in the outcomes was registered at least once: tests results went from positive to negative and from negative to positive again. The other 17 patients had only positive swabs and never a negative test result in our registry.

## 5. Conclusions

In our study, SARS-CoV-2 virus persistence in swabs reaches 63 days, with a median value of 30 days (±10.68). Moreover, lower C_T_ N values at the first positive swab are correlated with a longer persistence of qPCR positivity. Those findings suggest a virus coexistence with patients for long time periods, and that a longer recovery time is required when higher viral loads are measured. Nevertheless, to maximize the sensitivity, multiple tests should be performed, if possible, on different collected specimens. We also observed a reduction across the study period of the viral RNA load in naso-pharyngeal swabs, which is difficult to explain with the available information in the registry, but should be better investigated through the systematic and detailed collection of additional data for each positive individual about the clinical status and the possible way of exposure.

The situation on COVID-19 is constantly evolving, and new studies on SARS-CoV-2 are continuously becoming available. As a number of questions remain unanswered, new findings should be actively monitored, with surveillance activities adjusted accordingly.

## Figures and Tables

**Table 1:** Values of the Pearson’s correlation coefficients among the age of patients (*n* = 81), the C_T_ value of the first positive naso-pharyngeal swab, and the duration (days) of the positivity in repeated swabs in the same patient, overall and by gender. * Asterisks are denoting the significant values.

	Overall			Males			Females		
	**Age**	**Duration of Positivity**	**C_T_ Values**	**Age**	**Duration of Positivity**	**C_T_ Values**	**Age**	**Duration of Positivity**	**C_T_ Values**
**Age**	1	−0.077	−0.110	1	−0.167	−0.085	1	0.096	−0.144
**Duration of Positivity**	−0.077	1	−0.289 *	−0.167	1	−0.349 *	0.096	1	−0.183
**C_T_ Values**	−0.110	−0.289 *	1	−0.085	−0.349 *	1	−0.144	−0.183	1
